# Shear Performance of Reinforced Concrete Beams with Small Circular Openings Strengthened Using Rectangular and Octagonal-Shaped Reinforcement

**DOI:** 10.3390/ma13245804

**Published:** 2020-12-18

**Authors:** Hyeong-Gook Kim, Jung-Yoon Lee, Kil-Hee Kim

**Affiliations:** 1 Department of Architectural Engineering, Kongju National University, Cheonandaero, Seobuk, Cheonan 31080, Korea; anthk1333@kongju.ac.kr; 2 Department of Civil, Architectural, Environmental System Engineering, Sungkyunkwan University, 80, Deogyeongdaero, Jangan, Suwon 16419, Korea; jungyoon@skku.edu; 3 Department of Architectural Engineering and Urban Systems Engineering, Kongju National University, Cheonandaero, Seobuk, Cheonan 31080, Korea

**Keywords:** web opening reinforcement, cyclic loading, shear performance, crack control, strain distribution

## Abstract

An experimental case study was carried out to investigate the shear performance of reinforced concrete beams with small circular openings under a cyclic anti-symmetric bending moment. The openings were strengthened by using a newly developed reinforcement continuously bent into rectangular and octagonal shapes, which was convenient for installation and effective for crack control. The presence of web opening reinforcement, the reinforcing method, and the web opening spacing were employed as main variables in the design of five specimens. The cyclic performance of all specimens was evaluated in terms of failure mode, crack pattern, strength and stiffness degradation, and strain distribution. Experimental results were discussed to assess the suitability of the proposed web opening reinforcement in RC web opening beams. It was confirmed that the proposed web opening reinforcement exhibited outstanding crack control and served as a shear resistance component in place of the concrete cross-section lost due to web openings. Finally, the shear strength of all specimens, obtained from the cyclic loading tests, were compared with those obtained from the equation proposed by Mansur (1998) and the Architectural Institute of Japan standard 2010.

## 1. Introduction

A web opening beam system has the advantage of lowering the height of building structures, since utility ducts and pipes for electricity, air conditioning, water supply, and network cabling can be passed through the web openings. However, when reinforced concrete beams with web openings are used, the sudden change in beam cross-section can cause stress to concentrate around the web openings and result in diagonal cracks that affect beam strength. It is known that the cross-sectional loss of concrete due to web openings has a greater effect on beam shear strength than on flexural strength. When adequate reinforcement around the openings is not provided, beams with web openings located in a high-shear region experience a rapid decrease in strength due to premature cracking of concrete.

Research from the early 1960s to the early 1980s was focused on evaluating the structural performance of web opening beam systems in relation to the size, shape, position, arrangement, and the number of web openings [1,2,3,4,5]. From the late 1980s to 1990s, studies were conducted on design approaches [6,7,8,9] and predicting deflection and other behaviors of RC beams with web openings [10]. Following the development of analytical techniques and the establishment of related design codes, many experimental and analytical studies were performed on web opening size, shape, material strength, shear span-to-depth ratio, loading pattern, and the non-symmetry of beam cross-sections [11,12,13,14,15,16,17,18]. From the early 2000s to the present, researchers examined rectangular and circular web openings in terms of reinforcement using FRP (Fiber-Reinforced Plastics), GFRP (Glass Fiber-Reinforced Polymer), or CFRP (Carbon Fiber-Reinforced Polymer), and the effects of each approach [19,20,21,22,23,24,25,26]. 

When applying a web opening beam system to an RC structure, the usability of the structure may be affected by cracks and deflection around the web openings. To resolve this problem, some studies have proposed using web opening reinforcement for RC beams in the design stage, and a design approach based on such beams [9,27,28,29]. Some widely used design approaches for web opening beam systems with web opening reinforcement include the ACI 318-19 [30] and the AIJ standard 2010 [31], and the design equation proposed by Mansur (1998) [8]. The conventional web reinforcing method places shear or diagonal reinforcement near the web openings to control shear cracks cutting across the web openings. However, this method requires fixing the position of the reinforcement, and its poor constructability leads to a longer construction period and higher costs. Web opening reinforcement using FRP, GFRP, or CFRP sheets is also challenging since various factors such as sheet debonding measures, type, and directionality must be considered to reduce cracks and deflection. 

This study developed a web opening reinforcement that is continuously bent into rectangular and octagonal shapes; this is more convenient for installation because it is inserted between shear reinforcements. It also allows effective shear reinforcement and crack control in response to cross-sectional loss due to web openings. To evaluate the crack control effect, shear performance, and the behavior of the web opening beams, cyclic loading tests were performed. As variables, the tests were subject to anti-symmetric bending and shear in the presence of web opening reinforcement, reinforcing method, and web opening spacing. By comparing results for shear strength, crack patterns, stiffness degradation, and strain distribution in the RC beams in relation to the presence of web opening reinforcement, this study aimed to evaluate the suitability of the proposed web opening reinforcement for RC web opening beams. Based on comparisons between experimental and analytical results, this study investigated the feasibility of using existing design approaches suggested by Mansur [8] and the AIJ standard 2010 [31] to predict the shear strength of RC beams with the newly developed web opening reinforcement.

## 2. Experimental Program

### 2.1. Material Properties

The design strength of the concrete used to fabricate the specimens was set to be 24 MPa, and the concrete mix design is presented in Table 1. Portland cement type I was used to make the concrete specimens. Cylindrical concrete specimens with a diameter of 100 mm and a height of 200 mm were fabricated for property evaluation and cured in the same conditions as other specimens. A 28-day compressive strength test was performed on the concrete cylinders according to standard testing methods and procedures presented in ASTM (American Society for Testing and Materials) C39 [32]. The average compressive strength of the three cylindrical concrete specimens, f’_c_, was found to be 20.9 MPa on the day of testing.

D25 bars with a yield strength of 610 MPa were used so that all specimens experienced shear failure before the flexural yielding of the longitudinal reinforcement. D6 bars with a yield strength of 300 MPa were used for the web opening reinforcement. To prevent specimen strength from being determined by longitudinal reinforcement and concrete bond failure, U-shaped bars were installed on the inner longitudinal reinforcement at the same intervals as the shear reinforcement. D10 bars with a yield strength of 540 MPa were used as U-shaped bars and shear reinforcement. The mechanical properties are given in Table 2.

### 2.2. Details of the Web Opening Reinforcement

**Figure 1:**
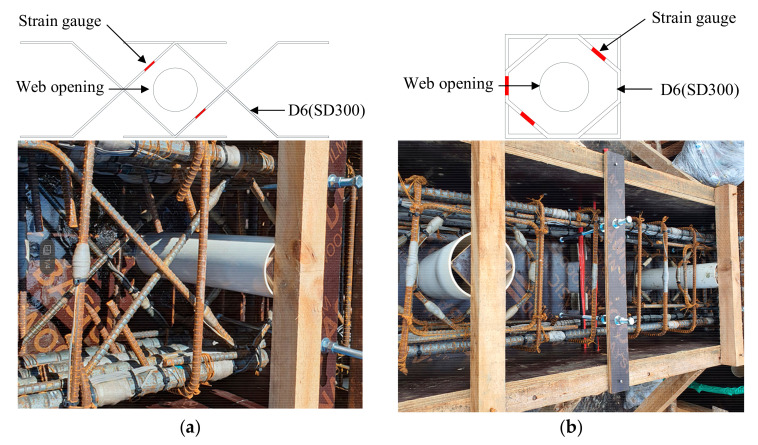
Details of web opening reinforcements: (**a**) Type A; (**b**) Type B.

This study developed a web opening reinforcement with new shapes to reinforce areas surrounding web openings and to improve constructability when arranging it in the field. Figure 1 shows details of an existing web opening reinforcement (type A) and of the web opening reinforcement (type B) proposed in this study. The web opening reinforcement comprises of a steel bar continuously bent into rectangular and octagonal shapes. The proposed web opening reinforcement uses less steel and is easier to fabricate than the web opening reinforcement by Kim et al. (2019) [33], which includes rectangles and rhombuses in a spiral configuration. The proposed reinforcement also offers better constructability because skilled workers can easily install the developed web opening reinforcement between shear reinforcements after arranging them as planned. For the web opening reinforcement shown in Figure 1b, the rectangular steel bar was designed to serve as shear reinforcement and to improve constructability, while the octagonal steel bar was designed to control diagonal cracks passing through web openings. The web opening reinforcement was fabricated to secure 40 mm of concrete cover at both ends of the web openings and was installed on both sides of the openings. 

### 2.3. Specimen Details

To evaluate the shear performance of the web opening beams in relation to the shape of the web opening reinforcement, the specimens were designed such that the shear reinforcement and web opening reinforcement will yield before the yielding of the longitudinal reinforcement. Table 3 shows the properties of the specimens. The main design variables were the presence of web openings and web opening reinforcement, the shape of the web opening reinforcement, and the web opening spacing. Hanson (1969), Some and Corley (1974) [34,35] found that when the size of circular openings is less than 30% of the beam depth, the shear strength of the beams does not decrease. They also noted that providing stirrups on either side of the opening may fully restore the strength of a beam with openings. In this study, openings with a diameter of h/3 larger than 30% of the beam depth were designed to evaluate the effect of the developed opening reinforcement. To prevent a decrease in the strength of beams due to concrete crushing, openings were arranged at three times the web opening diameter and at equal intervals from the center of a section excluding the distance from critical sections for shear and bending to the supports (375 mm). Moreover, considering the introduction of multiple openings into a beam, the spacing between openings was set to be two or three times the web opening diameter to ensure that proper spacing of the openings caused no reduction in the shear strength of beams, or any difference in the behavior of beams. To evaluate the utilization of beam span due to the introduced openings, four openings were installed in the beams based on the designed values of opening diameter and spacings.

**Figure 2:**
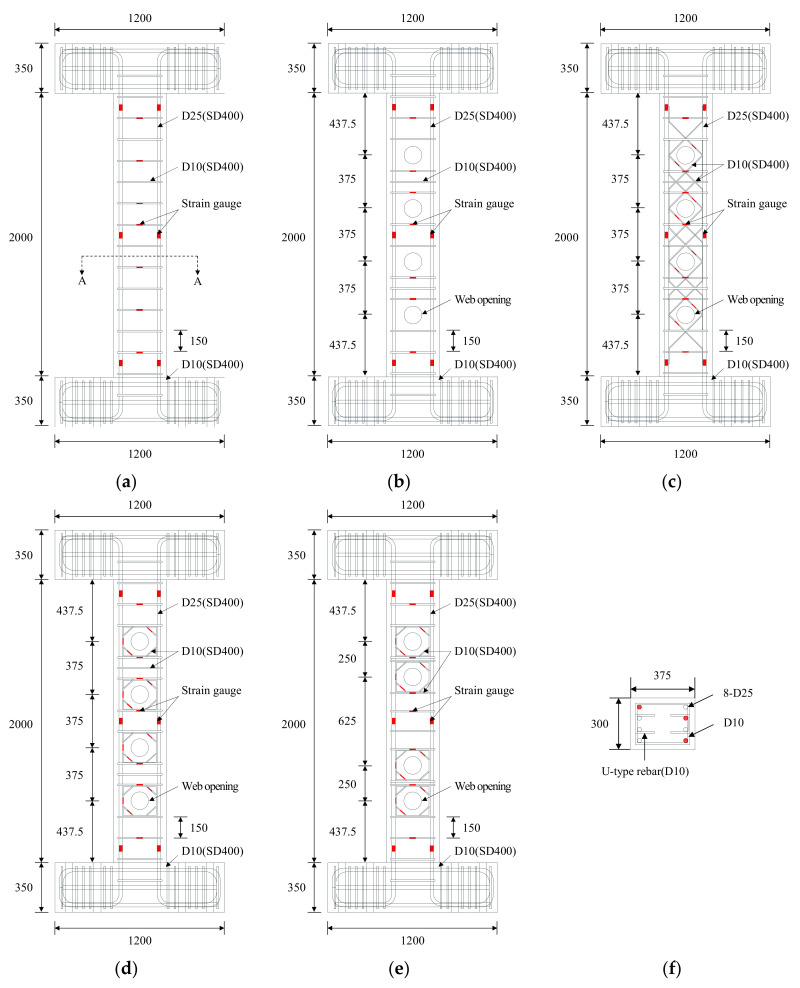
Details of specimens (unit: mm): (**a**) No. 1; (**b**) No. 2; (**c**) No. 3; (**d**) No. 4; (**e**) No. 5; (**f**) Section A-A.

Figure 2 presents the details of the specimens and the arrangement of web openings and web opening reinforcement. All specimens had a width (b) of 300 mm, height (h) of 375 mm, and a shear span-to-depth ratio (a/d) of 3.08. The specimen without web openings, No. 1, was set as the control specimen. Shear performance and behavior between the control specimen and other specimens were compared in relation to the presence of web openings and web opening reinforcement. The diameter of the web opening (Φ) was one-third of the effective depth (d), and the opening space was set to be two or three times the web opening diameter (Φ) to determine behavioral differences in relation to spacing. The types of web opening reinforcement include the existing type A used in No. 3 and the proposed type B used in No. 4 and No. 5. For all specimens, four D25 bars each were placed on tensile and compressive-side cross-sections as shown in Figure 2f for shear failure to precede other types of failure. D10 shear reinforcements were placed at a spacing of 150 mm. To prevent bond failure between the longitudinal reinforcement and concrete (Kim et al. 2014) [36], U-shaped reinforcements were introduced to the inner side of the longitudinal reinforcement.

### 2.4. Test Setup and Instrumentation

**Figure 3:**
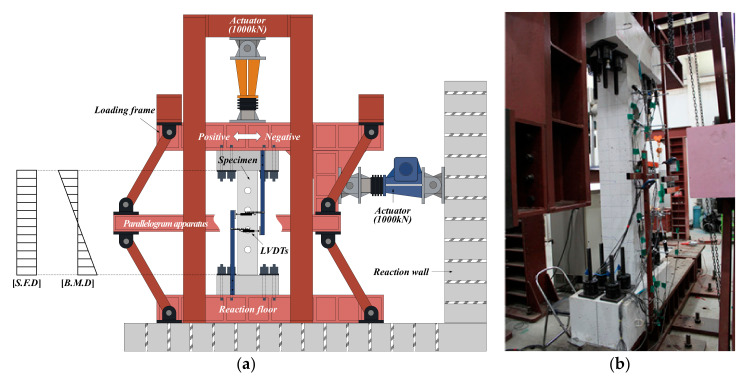
View of test setup: (**a**) schematic of test setup; (**b**) photo of experimental setup.

Figure 3 provides a view of the test setup. A parallelogram apparatus was used so that specimens would undergo only anti-symmetric moment without axial force due to weight, and a horizontal load was applied to the specimens using a 1000 kN actuator. A vertical actuator suspended between four steel columns was not used in the test because the specimens were fabricated to simulate RC moment-resisting frame beams. The specimens were fixed to the upper loading frame and the lower reaction floor with sixteen hydraulic nuts.

**Figure 4:**
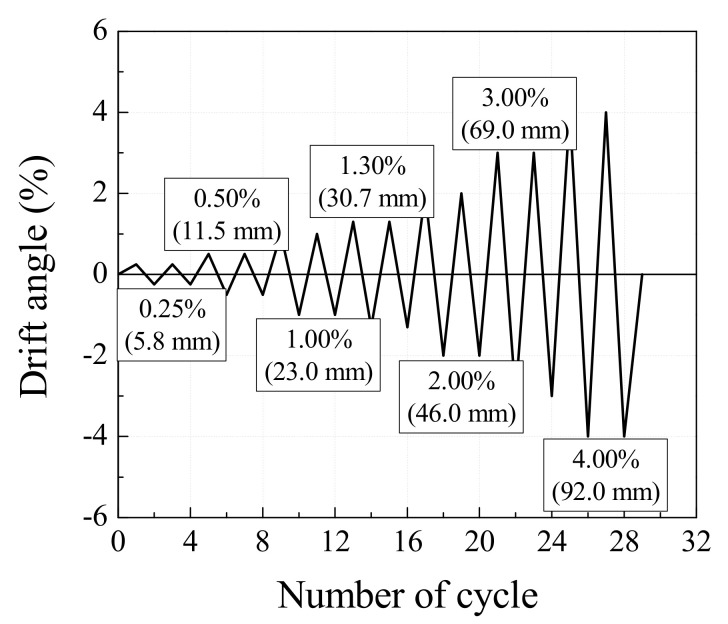
Loading history.

Figure 4 shows the loading history under the displacement control method, with two cycles repeated per loading step. The specimens were loaded monotonically according to the set loading history. Two Linear Variable Differential Transformers (LVDTs) with 300 mm capacity were attached to the vertical steel frames installed on the upper and lower stubs. The horizontal displacement, drift angle, and ductility of the specimens in positive and negative loading directions were obtained from the relative displacement between the two LVDTs. To monitor the yields of the longitudinal and transverse reinforcements during the test, strain gauges were attached at regular intervals to the reinforcements as shown in Figure 2. The location of strain gauges for type A and B web opening reinforcements can be found in Figure 1. Based on the recommendations outlined in the commentary to Section 2.8.1 of ASCE Std. 41-06 [37], testing protocols were planned to examine expected failure mode, change in stiffness, and behavior of specimens. Cyclic Loading was determined when the acting load was lowered to 85% of the maximum strength of the specimens in either loading direction.

## 3. Test results and Discussions

### 3.1. Load Versus Drift Angle Relations

**Figure 5:**
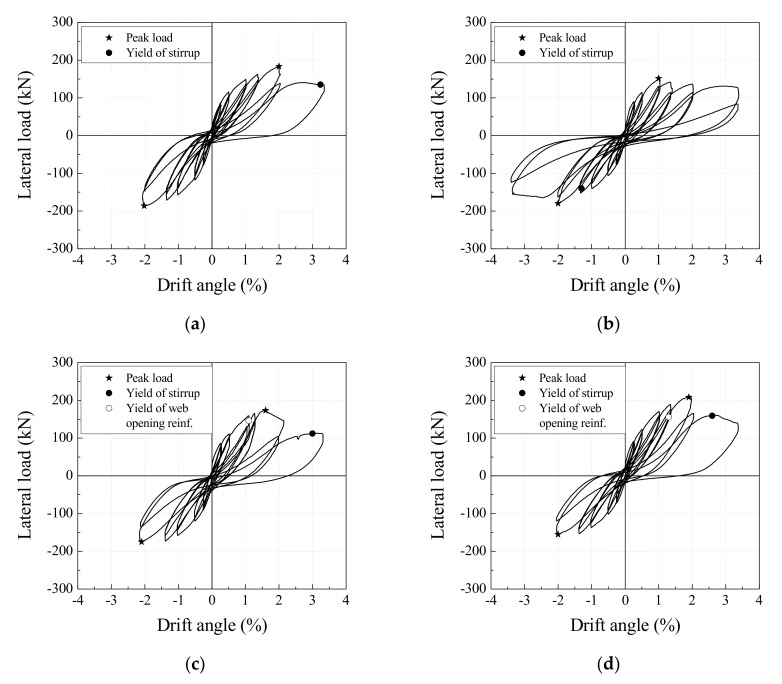

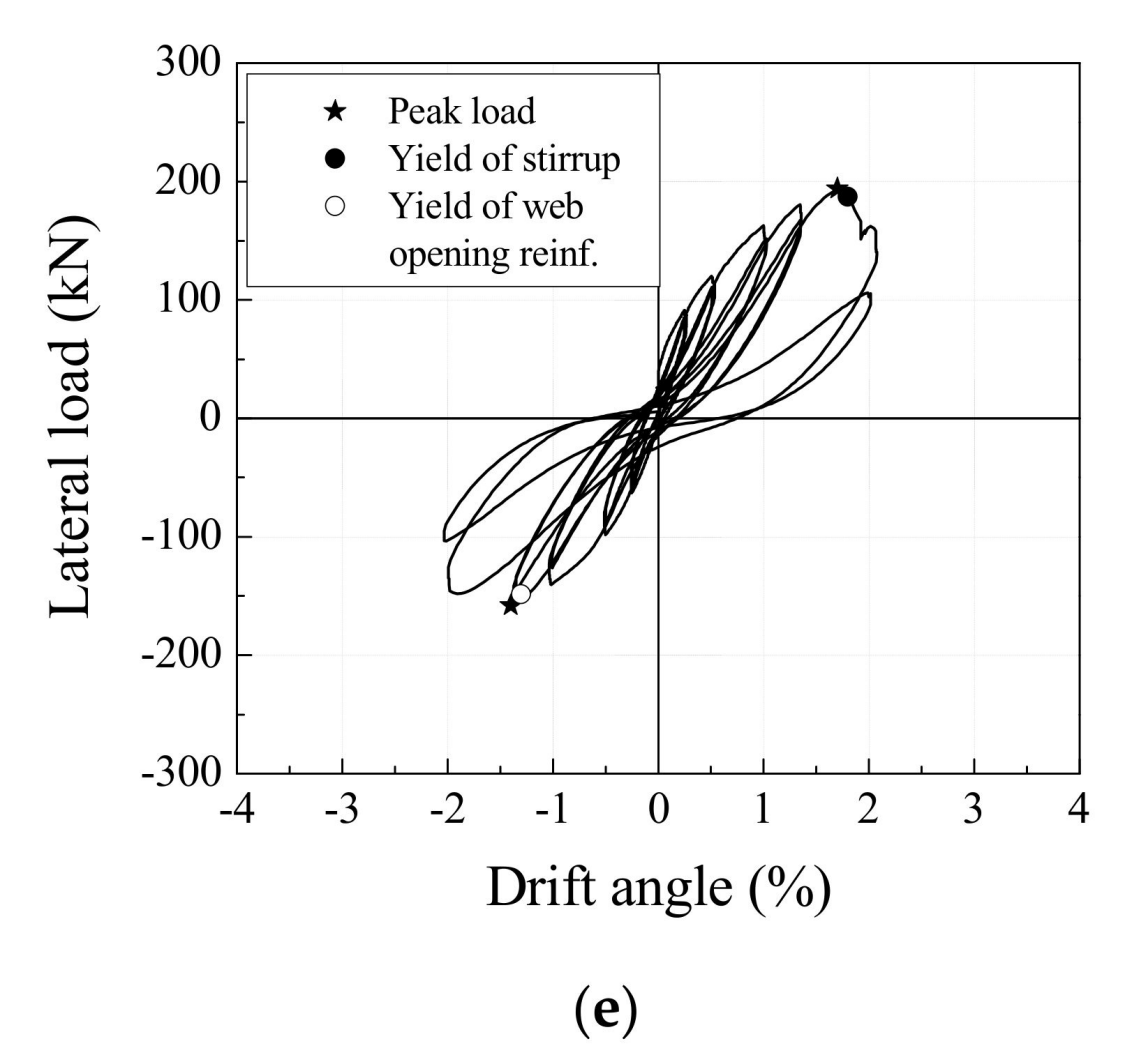
Lateral load-drift angle relations: (**a**) No. 1; (**b**) No. 2; (**c**) No. 3; (**d**) No. 4; (**e**) No. 5.

Figure 5 shows the load versus drift angle relations for each specimen. The yielding points of the shear reinforcement and the web opening reinforcement are specified. Table 4 provides a comparison of loads and drift angles at the yielding of shear and web opening reinforcements and at the maximum strength of specimens. The control specimen, No. 1, reached a maximum strength of −186.5 kN at a drift angle of −2.0% before the shear reinforcement yielded. Specimen No. 1 saw a decrease in strength to 85% of maximum strength due to bond splitting cracks that formed along with the longitudinal reinforcement after peak loading. It failed when the shear reinforcement yielded at a drift angle of 3.3%. Specimen No. 2, which had no reinforcement of web openings, experienced shear reinforcement yielding at a drift angle of −1.3% and reached a maximum strength of −179.8 kN at a drift angle of −2.0%. Specimen No. 2 failed at a drift angle of 3.3% due to the increasing width of diagonal cracks across the web openings and concrete crushing. Specimen No. 3, with web openings reinforced by the type A web opening reinforcement, experienced yielding of the diagonal reinforcement at a drift angle of 1.1% and reached the maximum strength of −175 kN at a drift angle of −2.1%. The strength of specimen No. 3 declined rapidly with the formation of diagonal cracks across the web openings, and shear reinforcement yielded at a drift angle of 3.0% after maximum strength.

Specimen No. 4, with web openings reinforced by type B web opening reinforcement, experienced yielding of web opening reinforcement and shear reinforcement at drift angles of 1.3% and 2.6%, respectively. Specimen No. 4 reached a maximum strength of 208.5 kN at a drift angle of 1.9%, and this was the highest strength among all specimens. Similar to specimen No. 3, it failed due to the yielding of the shear reinforcement, caused by diagonal cracks across the web openings. Specimen No. 5, with an opening space of 2Φ and web openings reinforced by type B web opening reinforcement, experienced web opening reinforcement yielding at a drift angle of −1.3%, and reached a maximum strength of 193.7 kN at a drift angle of 1.7%. The shear reinforcement yielded immediately after maximum strength, and shear failure was caused by diagonal cracks around the web openings and concrete crushing between web openings.

### 3.2. Crack Patterns and Failure Modes

**Figure 6:**
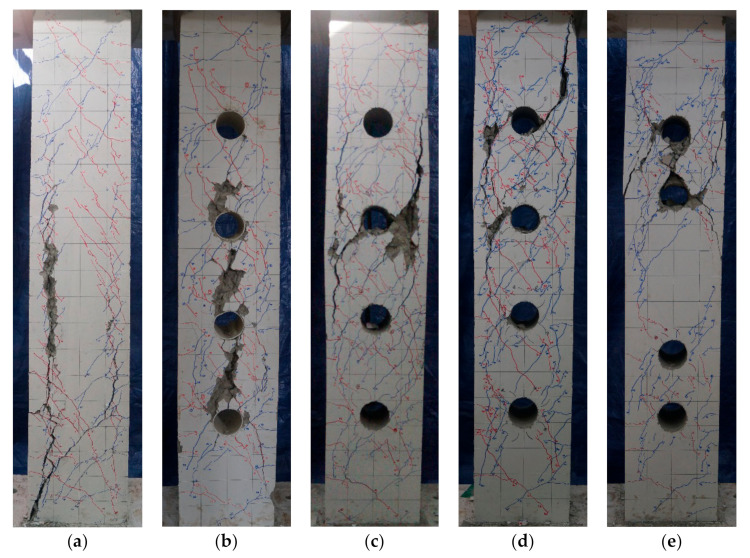
Crack patterns of specimens at failure: (**a**) No. 1; (**b**) No. 2; (**c**) No. 3; (**d**) No. 4; (**e**) No. 5.

Schematics of crack patterns of specimens at failure are shown in Figure 6. All specimens formed flexural cracks at both ends at a drift angle of 0.25% during initial loading and shear cracks at a drift angle of 0.5%. Specimen No. 1 experienced shear bond failure due to shear cracking at a drift angle of 2.0% after the formation of the initial flexural cracks, and the expansion of splitting cracks occurred along with the longitudinal reinforcement. Specimen No. 2 saw a significant increase in the width of shear cracks around the web openings with increasing load after the formation of flexural cracks and experienced shear failure due to concrete crushing resulting from the joining of shear cracks between web openings. Specimens No. 3 and No. 4 experienced cracks similar to those of specimen No. 2 during initial loading; however, the number of cracks rather than the crack width surged with increasing load. Unlike specimen No. 2, the specimens experienced shear failure due to the yielding of the shear reinforcement and web opening reinforcement under higher loads. Specimen No. 5 also exhibited crack patterns similar to specimens No. 2, No. 3, and No. 4 during initial loading, but experienced earlier yielding of the shear reinforcement and web opening reinforcement than specimens No. 3 and No. 4, because the cracks were concentrated on narrower concrete cross-sections between web openings.

### 3.3. Effective Shear Stiffness

**Figure 7:**
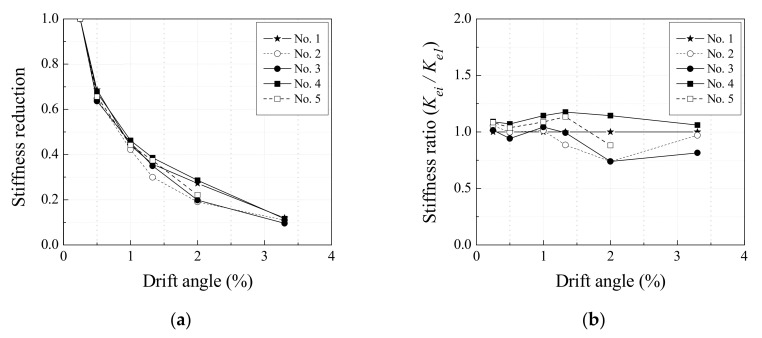
Effective stiffness of specimens with respect to drift angle: (**a**) stiffness reduction; (**b**) stiffness ratio.

Figure 7a shows the reduction in effective shear stiffness of each specimen with respect to drift angle in a positive direction. The effective shear stiffness is defined here as the ratio of the strength to the drift angle. While the effective shear stiffness of specimen No. 4 decreased at a similar gradient to specimen No. 1 at a drift angle between 1% and 2%, this was less drastic compared to the decrease in the effective shear stiffness of specimens No. 2, No. 3, and No. 5. Figure 7b shows the ratio of effective shear stiffness ($K_{ei}$) of each specimen to the effective shear stiffness ($K_{e1}$) of specimen No. 1 with respect to drift angle in a positive direction. Up to a drift angle of 2.0%, specimens No. 2 and No. 3 had a lower effective shear stiffness than specimen No. 1, and the effective shear stiffness of specimen No. 5 decreased at a gradient similar to specimen No. 3 after a drift angle of 1.33% due to concrete crushing around web openings. On the other hand, specimen No. 4 maintained a high effective shear stiffness, higher than specimen No. 1 by 14% on average, even after maximum strength.

Many experimental results are needed to determine the optimal size of web openings and spacing between web openings and ensure the stiffness of RC web opening beams and shear performance. This study verified that RC web opening beams reinforced with the proposed web opening reinforcement had similar or better shear performance compared to RC beams without web openings when web openings were spaced at least 3Φ apart, and that the proposed reinforcement was more effective than existing web opening reinforcements for effective shear stiffness and shear strength.

### 3.4. Effect of Shape of Web Opening Reinforcement

**Figure 8:**
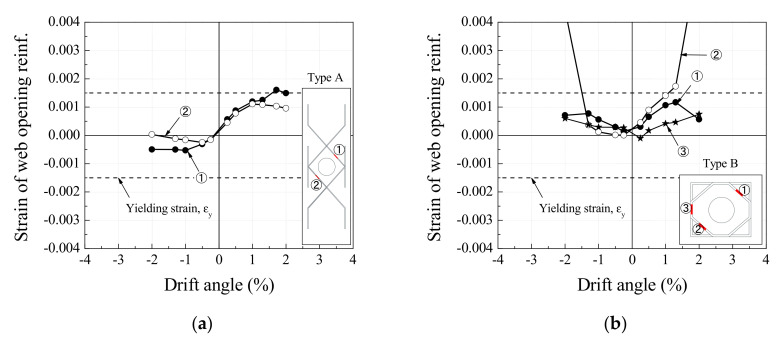

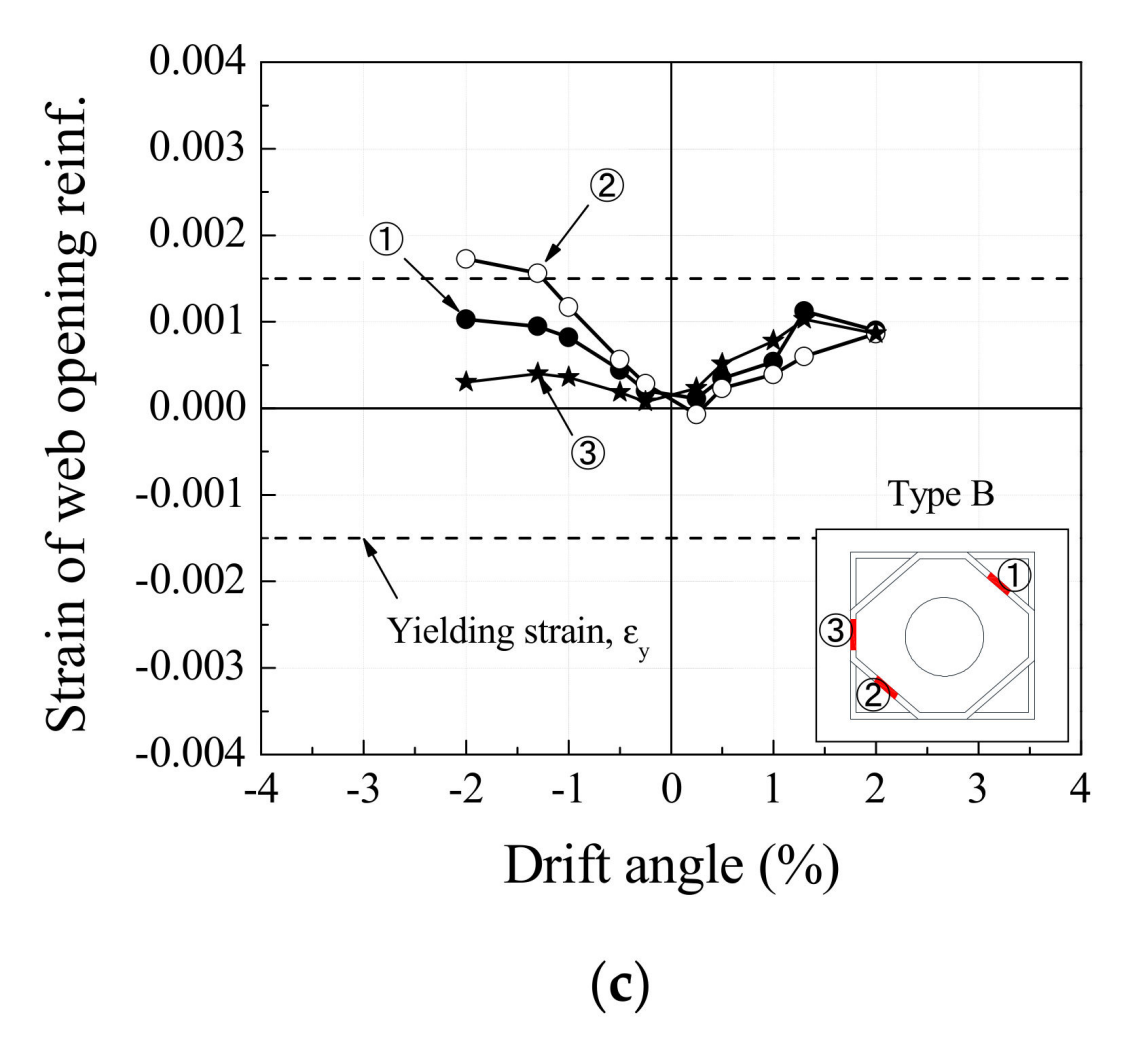
Strain of web opening reinforcement with respect to drift angle: (**a**) No. 3; (**b**) No. 4; (**c**) No. 5.

Figure 8 shows the strain distribution in the web opening reinforcements installed to reinforce web openings with respect to drift angle. The shape of the web opening reinforcement and the positions of the strain gauges are marked in the same figure. Figure 8a represents that ① and ② of the type A web opening reinforcement resisted tension under drift angles in the positive direction, and compressive force under drift angles in the negative direction. This implies that the diagonal bars placed across shear cracks in each direction were the only elements resisting shear cracks. On the other hand, ① to ③ of the type B web opening reinforcement was installed for specimens No. 4 and No. 5 resisted tension for drift angles in both the positive and negative directions. It can be inferred from Figure 8b,c that the web opening reinforcement without strain gauges resisted tension, unlike the type A web opening reinforcement.

Based on the yielding points of the web opening reinforcement and shear reinforcement shown in Figure 5, and the strain distribution in Figure 8, the proposed web opening reinforcement was confirmed to be more effective than the type A web opening reinforcement for controlling cracks in all directions surrounding the web openings. It enhanced the shear strength of the RC web opening beams by resisting shear cracks, in place of the smaller concrete cross-section, due to web openings.

### 3.5. Effect of Web Opening Spacing

**Figure 9:**
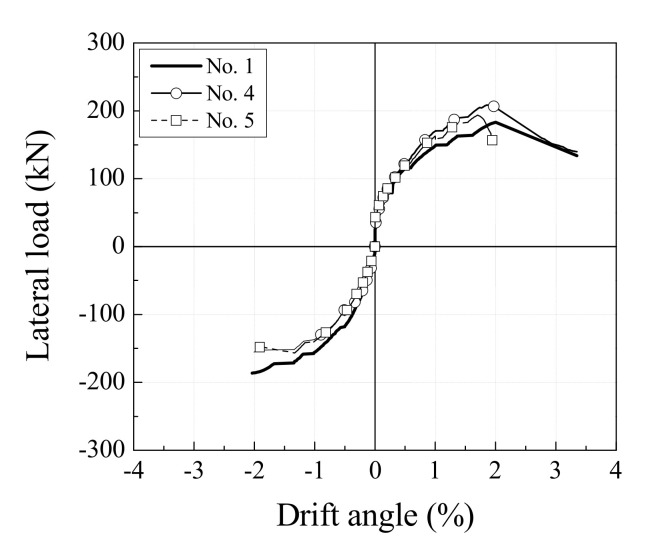
Effect of spacing of web opening on ultimate load and ductility.

Figure 9 compares the load-drift angle envelope curves of specimens No. 1, No. 4, and No. 5. Specimens with web opening reinforcement, regardless of type, saw an increase in maximum strength and ductility relative to specimen No. 2. In particular, the maximum strength of specimen No. 4 occurred at a drift angle in a positive direction. The strength of specimen No. 5, with an opening space of 2Φ, was lower than that of specimen No. 4 by about 9.3%. Specimen No. 5 failed at the smallest drift angle. While its maximum strength was higher than that of specimen No. 1, the specimen experienced a rapid decrease in load after maximum strength due to the significant increase in the width of cracks between web openings and exhibited brittle behavior. The results show that the opening space should be at least 3Φ since a narrower opening space of 2Φ caused a rapid decrease in strength because of the reduction in the shear resistance components in the web area.

## 4. Prediction of Shear Strength of RC Beam with Small Web Openings

**Figure 10:**
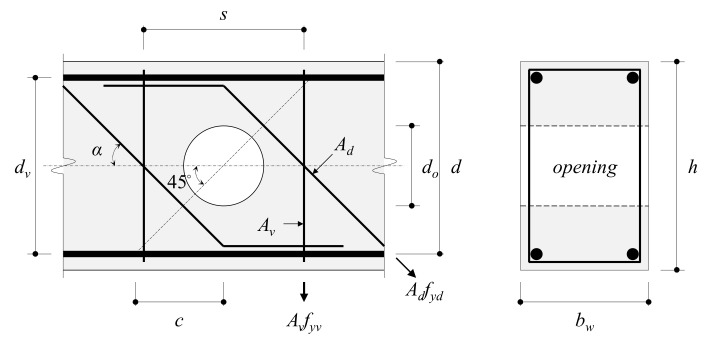
Shear resistance, V_s_, provided by shear and diagonal reinforcements at a single opening (AIJ 2010).

This section compares the shear strength prediction for RC beams with small web openings using the equation proposed by Mansur (1998) and the AIJ standard 2010. Mansur found that web openings between the depth of the equivalent rectangular stress block in negative and positive directions do not significantly influence the flexural behavior of beams. However, they highlighted the need to review the effects of web opening position and size on shear strength and behavior when beams were subject to significant shear forces, which can cause a decrease in shear resistance components ($V_{c}$) with the loss of concrete cross-sections. The equation by Mansur (1998) and the AIJ standard 2010 calculate the shear strength of RC beams having web openings ($V_{n}$) as the sum of the shear resistance component of concrete ($V_{c}$) and the shear resistance component of shear reinforcement ($V_{s}$). Figure 10 shows the shear resistance, $V_{s}$, provided by transverse and diagonal reinforcements at a single opening.

Mansur (1998) proposed the following equation to calculate the shear strength ($V_{n.M}$) of RC beams with small web openings.

(1) $$V_{n.M}=V_{c}+V_{s}$$

(2) $$V_{c}=\left[\frac{1}{6}\sqrt{f_{c}^{\prime }}\left(d-d_{o}\right)\right]b_{w}$$

(3) $$V_{s}=V_{sv}+V_{sd}=\frac{A_{v}f_{yt}}{s}\left(d_{v}-d_{o}\right)+A_{d}f_{yd}\sin\alpha$$

Here, $f_{c}^{\prime }$: compressive strength of concrete (MPa); $d_{o}$: diameter of the web opening (mm); d: effective depth of the beam (mm); $d_{v}$: distance between the top and bottom longitudinal reinforcement (mm); $b_{w}$: beam width (mm); $V_{sv}$: shear resistance component of the transverse reinforcement (N); $V_{sd}$: shear resistance component of the diagonal reinforcement (N); $A_{v}$: cross-sectional area of transverse reinforcement (${\mathrm{mm}}^{2}$); $f_{yt}$: yield strength of transverse reinforcement (MPa); s: spacing of transverse reinforcement (mm); $A_{d}$: area of diagonal reinforcement of the failed surface (${\mathrm{mm}}^{2}$); $f_{yd}$: yield strength of the diagonal reinforcement (MPa); α: angle of the diagonal reinforcement (rad).

According to the AIJ standard 2010, the shear strength of RC beams with small web openings ($V_{n.AIJ}$), shear resistance component of concrete ($V_{c}$), and shear resistance component of shear reinforcement ($V_{s}$) can be calculated as follows.

(4) $$V_{n.AIJ}=V_{c}+V_{s}$$

(5) $$V_{c}=\left[\frac{0.092k_{u}k_{p}\left(f_{c}^{\prime }+17.7\right)}{\frac{M}{Vd}+0.12}\left(1-\frac{1.61d_{o}}{h}\right)\right]b_{w}d_{v}$$

(6) $$V_{s}=0.846\sqrt{\rho_{w}f_{yt}}b_{w}d_{v}$$

Here, $k_{p}$: coefficient ($=2.36{\left(\rho_{t}\right)}^{0.23}$) considering the effect of tension reinforcement ratio ($\rho_{t}$); $k_{u}$: coefficient (0.72~1.0) considering the size effect of the effective depth (d) of the beam; M: flexural moment acting on the beam (N⋅mm); V: shear force acting on the beam (N); h: height of the beam (mm). 

$\rho_{w}$, the ratio of web opening reinforcement within $d_{v}/2$ from the center of web openings, is calculated by Equation (7).

(7) $$\rho_{w}=\frac{A_{v}\left(\sin\alpha +\cos\alpha \right)}{b_{w}c}$$

Here, c is $d_{v}/2$.

Table 5 compares the experimental and analytical results obtained using Equations (1) and (4). Mansur’s equation predicted the experimental results with an average accuracy of 0.85 and a coefficient of variation of 9.83%, while the AIJ standard 2010 predicted experimental results with an average accuracy of 1.05 and a coefficient of variation of 18.7%.

Compared to Equation (4), Equation (1) overestimated the shear strength of all specimens by 15% on average. This is because Mansur’s equation presents the nominal shear strength ($V_{n}$) of the web opening beams based on the shear design of typical RC beams, whereas the AIJ standard 2010 assumes that the concrete and steel bars will resist shear and represents the shear strength of the web opening beams including a safety factor based on the experimental results for tensile reinforcement ratio and size effect. However, like the AIJ standard 2010, Mansur’s equation is expected to provide rational predictions for the shear strength of RC web opening beams if a strength reduction coefficient is introduced in Equations (2) and (3), in consideration of the decrease in the bond stress of the tensile reinforcement, due to loss of the concrete cross-section caused by the web openings and the size effect of members.

## 5. Conclusions

This study performed experiments on crack control of RC web opening beams with square and octagonal-shaped web opening reinforcement and evaluated the shear performance of RC web opening beams using the proposed web opening reinforcement under cyclic loading. The following conclusions were derived from the experimental study.

- With the proposed web opening reinforcement, the RC web opening beams had similar or better shear strength and shear behavior, compared to RC beams without web openings and RC web opening beams with the existing bent-type web opening reinforcement. They maintained an effective shear stiffness that was 11% higher on average, even with an increase in member deformation.
- It was observed that the crack patterns around openings strengthened using the existing and proposed web opening reinforcements are almost identical, although the proposed web opening reinforcement has an advantage, compared to the existing method, of reducing the time for arranging reinforcement. However, unlike the existing bent-type web opening reinforcement, the proposed web opening reinforcement tends to resist tension for drift angles in both the positive and negative directions. The proposed web opening reinforcement did not experience concrete crushing around the web openings, even under extreme conditions, thanks to outstanding crack control, and served as a shear resistance component in place of the concrete cross-section lost due to web openings.
- If the spacing between web openings is smaller than three times the opening diameter, the decrease in concrete cross-section that is needed for stress transfer of beams by arch action can cause a rapid decline in member strength.
- Compared to the equation by Mansur (1998), the AIJ standard 2010 provided more rational predictions for the shear strength of the RC web opening beams using the proposed web opening reinforcement. However, Mansur’s equation is expected to present sufficiently rational and stable predictions if a strength reduction coefficient is introduced to reflect the decrease in the bond stress of the tensile reinforcement in relation to web opening position, the size effect of members, and the loss of concrete cross-section caused by web openings.
- The results of experiments and analysis showed that the diameter of circular openings should be less than one-third of the effective depth of the RC beams, and openings should be in the section (L-2D) excluding the distance from the critical section for shear (D) to the support to prevent the decrease in the shear strength of the beams with web openings. Moreover, it is recommended that the spacing between web openings should be determined based on the structural calculation results but should be at least three times the opening diameter when multiple openings are installed.

## Figures and Tables

**Table 1:** Concrete mix design.

f_ck_	G_max_	W/C	S/a	Unit Weight (kg/m^3^)				
(MPa)	(mm)	(%)	(%)	W	C	S	G	AD
24	25	49.7	48.5	82	214	872	936	69.3

f_ck_: compressive strength of concrete mix design, G_max_: maximum size of coarse aggregate, W/C: water cement ratio, S/a: fine aggregate modulus, W: water, C: cement, S: fine aggregate, G: coarse aggregate, and AD: water-reducing admixture.

**Table 2:** Mechanical properties of steel bars.

Bars	Diameter (mm^2^)	A_s_ (mm^2^)	f_y_ (MPa)	f_u_ (MPa)	ε_y_ (-)	ε_u_ (-)	Position of Steel Bars
D6	6.53	31.6	300	320	0.0015	0.0371	Web opening reinforcement
D10	9.53	71.3	540	639	0.0027	0.0823	Shear rebar and U-shaped bar
D25	22.2	387.1	610	750	0.0031	0.0792	Longitudinal reinforcement

A_s_: cross-sectional area, f_y_: yield strength of reinforcement, ε_y_: yield strain of reinforcement.

**Table 3:** Properties of specimens.

Specimen	b (mm)	h (mm)	a/d (-)	Longitudinal Reinf.	Diameter of Opening, Φ (mm)	Opening Space	Type of Web Reinf.
No. 1	300	375	3.08	8D-25	-	-	-
No. 2				8D-25	125 (h/3)	3Φ	-
No. 3				8D-25	125 (h/3)	3Φ	Type A (D6)
No. 4				8D-25	125 (h/3)	3Φ	Type B (D6)
No. 5				8D-25	125 (h/3)	2Φ	Type B (D6)

**Table 4:** Summary of experimental results.

Specimen	Yielding of Web Reinf.		Yielding of Shear Reinf.		Peak Load		Failure Mode
	Load (kN)	Drift Angle (%)	Load (kN)	Drift Angle (%)	Load (kN)	Drift Angle (%)	
No. 1	-	-	-	-	183.2	2.0	Shear
					−186.5	−2.0	
No. 2	-	-	−140.2	−1.3	151.9	1.0	Shear
					−179.8	−2.0	
No. 3	146.0	1.1	112.3	3.0	173.5	1.6	Shear
					−175.1	−2.1	
No. 4	155.5	1.3	159.3	2.6	208.5	1.9	Shear
					−155.4	−2.0	
No. 5	−148.6	−1.3	187.1	1.8	193.7	1.7	Shear
					−158.2	−1.4	

**Table 5:** Comparison between experimental and analytical results.

Specimen	Experimental Results		Analytical Results		*V_n.E_*/*V_n.M_*	*V_n.E_*/*V_n.AIJ_*
	Drift Angle (%)	*V_n.E_* (kN)	*V_n.M_* (kN)	*V_n.AIJ_* (kN)		
No. 1	2.0	183.2	215.5	161.1	0.85	1.14
	−2.0	−186.5			0.87	1.16
No. 2	1.0	151.9	187.0	126.1	0.81	1.20
	−2.0	−179.8			0.96	1.43
No. 3	1.6	173.5	200.4	180.1	0.87	0.96
	−2.1	−175.1			0.87	0.97
No. 4	1.9	208.5	219.3	199.4	0.95	1.05
	−2.0	−155.4			0.71	0.78
No. 5	1.7	193.7	219.3	199.4	0.88	0.97
	−1.4	−158.2			0.72	0.79
Ave.	-				0.85	1.05
CV	-				0.10	0.19

*V_n.E_*: Shear strength of specimen, *V_n.M_*: Analytical shear strength obtained from the equation proposed by Mansur (1998), *V_n.AIJ_*: Analytical shear strength obtained from the equation given in AIJ 2010 code.
